# Experimental and Numerical Research on Open-Hole Strength and Damage Mechanism of Regularly Arrayed Short Fiber Reinforced Polymer Composite

**DOI:** 10.3390/polym12071622

**Published:** 2020-07-21

**Authors:** Junfeng Hu, Xutong Zhang, Zhou Chen, Wenkang Guo, Hang Li, Xi Deng

**Affiliations:** 1 School of Mechanical and Power Engineering, Nanjing Tech University, No. 30 Pu Zhu South Road, Nanjing 211816, China; zhxtchina@163.com (X.Z.); zchen6240@njtech.edu.cn (Z.C.); Wenkang_Guo@163.com (W.G.); 2 College of Civil Engineering, Hunan University, Yuelu Mountain, Changsha 410082, China; rikou@hnu.edu.cn; 3 School of Materials Science and Engineering, South China University of Technology, No. 381 Wushan Road, Tianhe District, Guangzhou 510641, China

**Keywords:** open hole strength, UACS laminate, tensile test, finite element method

## Abstract

Laminates with unidirectionally arrayed chopped strands (UACS) are one of the advanced short fiber reinforced polymer composites (SFRP) with significant application prospect, which greatly improves mechanical properties compared to the traditional SFRP, meanwhile ensuring excellent flowability. In practice, composite laminate with an open hole is one of the typical connective components, and it is necessary to clarify the allowable load and damage tolerance performance of notched structures. In the present study, UACS laminates were fabricated using the continuous carbon fiber reinforced polymer (CFRP) prepreg, on which regularly arrayed bi-angled slits were introduced by a commercial numerical control cutter. The tensile strength and strain distribution around the open hole of the notched UACS laminate were experimentally investigated, while the damage progression near the open hole of the notched UACS laminate was analyzed by the finite element method (FEM). The tensile strength of the notched UACS laminate was measured at 298 MPa, which is about 60% of the strength of the unnotched UACS laminate. The simulation results match well with the experimental results, although there is a little overestimate on strength, by about 5% and 7%, for unnotched and notched UACS laminates, respectively. The final critical failure mode for the notched UACS laminate is mainly dominated by the delamination instead of the fiber breakage in the unnotched UACS laminate.

## 1. Introduction

Carbon fiber reinforced polymer (CFRP) composite material is widely applied in aerospace, automotive industry, wind power industry, and other fields, due to its series of advantages, such as light weight, high modulus and strength, low thermal expansion coefficient, etc. [1]. In addition, since vehicle fuel consumes nearly 70 percent of refined oil each year, in addition to the rapid growth in the number of vehicles in the world, it is difficult for energy development to meet the growing energy demand and also brings the environment problem caused by automobile exhaust emissions [2]. One of the most effective ways to reduce automobile fuel consumption and emission is through lightweight technology, such as using high-performance CFRP as manufacturing material for structures and components of vehicles instead of traditional metal materials [3].

However, the traditional continuous CFRP has some limitations in its application to components with complicated geometries, since it is prone to having heterogeneous distribution of fiber and resin in the curing process of fabricating complex structures. This defect will inevitably cause the nonuniformity of stress distribution in the material, affecting the structural performance, and, finally, become the potential hazard to the safety of the structure. To solve this problem, many researchers began to study short fiber reinforced polymer composites (SFRP) with excellent fiber flowability, such as sheet molded plastics (SMC), which has excellent fiber flowability and low cost [4,5,6]. Nevertheless, the strength and stiffness of components fabricated from SMC are far lower than the traditional continuous CFRP due to its low volume content of SMC fiber, the random distribution of fiber direction, and the uncontrollable fiber length.

**Figure 1:**
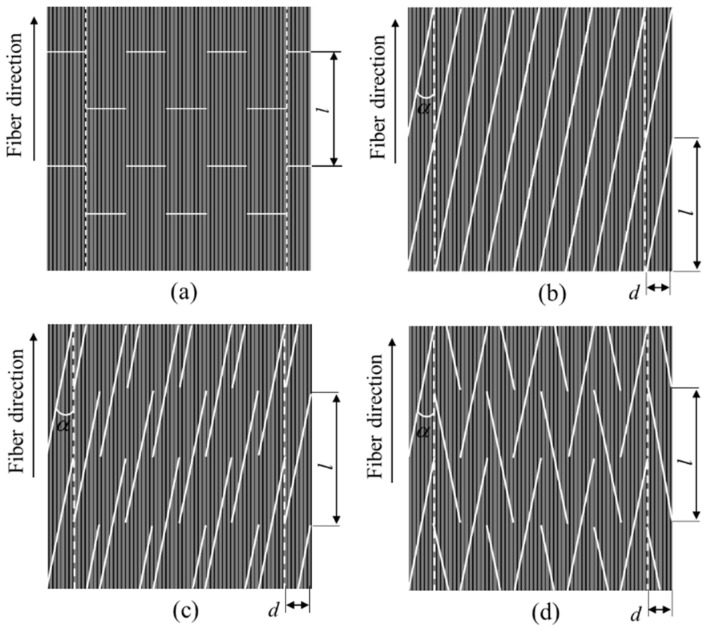
Existing types of slit patterns: (**a**) vertical slit [7], (**b**) continuous angled slit [8], (**c**) staggered-slit [10], (**d**) bi-angled slit [10].

In order to further improve mechanical properties of SFRP, researchers tried to develop a SFRP with unidirectional fiber distribution. Taketa proposed a new type of short fiber reinforced polymer composites UACS (Unidirectionally Arrayed Chopped Strands), as shown in Figure 1a, which was achieved by introducing series of regular slits in the traditional CFRP prepreg [7]. UACS exhibits better strength than former SFRP, while maintaining excellent fiber flowability, hence making it suitable for manufacturing load bearing structures with complicated geometries. As the direction of initial slits is perpendicular to the fiber direction, the interlaminar shear strength of CFRP laminate is much lower than the tensile strength in the fiber direction; it is easy to cause failure in slits at low load and lead to a large area of delamination damage. Therefore, the full utilization of high strength properties of carbon fiber is not satisfactory for this kind of UACS. After that, Taketa et al. improved the design scheme of the initial UACS and proposed the continuous slit with a small angle (Figure 1b) [8,9]. The proposed angled continuous slit reduced the normal stress at the slit compared to the initial UACS laminate under the same tensile load, thus improving the strength of the UACS laminate. However, since all the continuous slits are throughout the prepreg, once the delamination damage occurs somewhere in the slit, it will propagate along the slit direction and result in the final failure.

For the sake of inhibiting the occurrence and propagation of the interlayer damage in UACS laminates, and further improving the strength of UACS, Li and Wang proposed two kinds of discontinuous slits with a small angle, named as staggered-slit and bi-angled slit [10,11], as given in Figure 1c,d. Experimental results show that the tensile strength of UACS laminates with staggered-slits and bi-angled slits increased by 10% and 15% compared to the one with continuous slits and is 64% and 67% of the strength of traditional CFRP laminate without slits. The result of flowability testing showed that the dimension of the specimen along the fiber direction increased by 20% for the single layer UACS laminate with staggered-slits and bi-angled slits under the pressure of 2 MPa. Arifin and Wang further researched the static compression property of UACS laminates with staggered-slits and bi-angled slits and found that the energy absorption of newly proposed UACS laminates improved about 30.2% and 49.1%, respectively, compared to the traditional CFRP laminate [12]. Based on these research works, it could be concluded that UACS laminates with bi-angled slits have the best mechanical properties in the present SFRP composites, as reported so far. As mentioned above, the lightweight technology using high performance CFRP is one of the best ways to reduce the automobile fuel consumption and emission. Scholars have studied the application of CFRP composites in automobile collision prevention and other structures [13,14]. Therefore, it is significantly meaningful in the application of UACS laminates as the material of automobile structures with complicated geometries, instead of the traditional continuous prepreg.

However, most composite structures need openings or other notches for installation and assemblage in practical engineering problems. Composite laminates with an open hole are one of the typical connective components, and it is necessary to clarify the allowable load and damage tolerance performance of the structure with an open hole before any practical application because the notched area not only cuts off the continuous fiber but also causes the stress concentration around the notch. Researchers have done lots of work on the residual strength, failure mode, and failure mechanism of notched composite laminates. The thickness effect on the translaminar fracture toughness and strength of composites laminates with an open hole was investigated by Chen et al. through numerical method, which could capture the failure mode and patterns [15]. In order to investigate the strength of bolt connected CFRP laminates, experimental and numerical analyses were performed to clarify the stiffness and failure behavior of notched laminates [16]. Bao and Liu proposed a model based on the surface cohesive element for the progressive failure analysis of splitting and delamination for composite laminates with an open hole, and the simulation was well consistent with experimental results on strength and failure mode [17]. Du et al. studied the tensile progressive damage and failure mechanisms for a thermoplastic-based fiber metal laminate by the numerical method, which used three failure criterions of cohesive model, progressive, and ductile damage, and experimental results showed good agreement with numerical simulation [18]. Similarly, progressive damage models are established using continuum shell and cohesive elements to predict the strength of composite laminate with an open hole under compression [19,20]. It was also reported that different moisture and heat conditions were applied to study the environmental influence on strength and stiffness of laminates with an open hole, and a prediction method was developed based on the experimental data [21]. Some scholars also tried to detect the progressive damage and the final failure mode of notched CFRP laminates in the loading process by means of macro photography [22,23], digital image correlation [24], scanning electron microscope (SEM), and X-ray scanning equipment [25].

It could be easily inferred that the failure mechanism is quite complicated for UACS laminates compared to traditional continuous CFRP composites because a large number of discontinuous slits exist in UACS laminates. Therefore, it is necessary to clarify mechanical properties and failure mechanism of UACS laminates with an open hole under different load conditions, as well as the relationship between the strength and the size of open holes, before the wide application of UACS laminates. As seen from previous research [7,8,9,10,11,12], it could be concluded that UACS laminates with bi-angled slits have the best mechanical properties, including tensile strength, compression strength, specific energy absorption, and flowability during curing, among the existing four kinds of UACS laminates. Therefore, in this study, open-hole UACS laminates with regularly arrayed bi-angled slits were fabricated based on the continuous CFRP prepreg using a commercial numerical control cutter. The tensile strength and fracture morphology of notched UACS laminates, as well as the change of strain field around the open hole, were studied experimentally. Meanwhile, the tensile strength and modulus, critical failure loads for different types of damages, and the progressive failure process of the notched UACS laminate were investigated by the finite element method, which precisely simulated the distribution of slits.

## 2. Experimental and Simulation 

### 2.1. Materials and Fabrication

**Figure 2:**
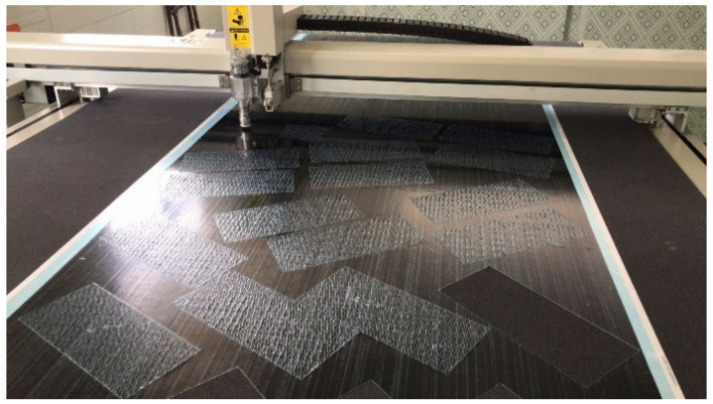
A commercial numerical control cutter.

**Figure 3:**
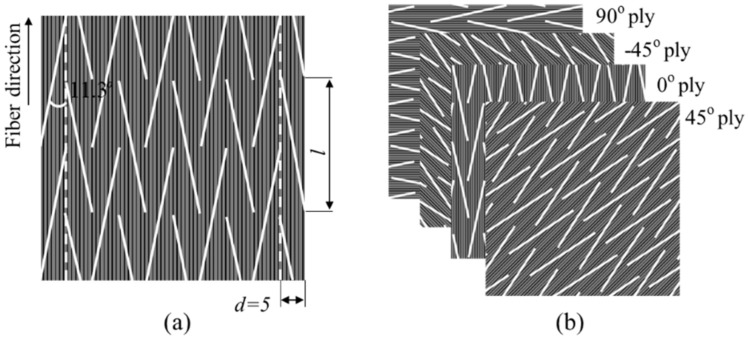
Slits and unidirectionally arrayed chopped strands (UACS) laminate: (**a**) bi-angled slit pattern, (**b**) stacking sequence.

In this research, the commercial continuous CFRP prepreg of UIN12500 (T800) (Guangwei, Weihai, China) was selected as the material, with the thickness of 0.125 mm and the fiber volume fraction of 60%. The longitudinal modulus, strength, and Poisson’s ratio of the prepreg are 139 GPa, 2900 MPa, 0.3, respectively. Bi-angled slits on the UACS prepreg, as well as prepregs for all the plies with different angles, were made by a commercial numerical control cutter (Jingwei, Ningbo, China) as shown in Figure 2. The schematic and dimension of bi-angled slits is shown in Figure 3a. The angle (α) between the slit and fiber direction, as well as the width of the slit (*d*), are set as 11.3° and 5 mm, based on the reported research [10,11]. It is easily imagined that the bi-angled slit keeps the orthotropic symmetry of received prepregs and is convenient for design and fabrication.

Specimens for tensile tests were from quasi-isotropic UACS laminates, which were fabricated with the stacking sequence of [45/0/-45/90]_s_ using prepregs with bi-angled slits, as depicted in Figure 3b. Stacked laminates are then cured by a mini test hot pressing machine (Toyo Seiki, Takarazuka, Japan) and a vacuum pump (Becker, Wuppertal, Germany). The curing process is controlled at the temperature of 130^o^ and the pressure of 0.4 MPa, according to the curing curve provided by the manufacturer of prepreg. It should be noted that the combination of mini test hot pressing machine and vacuum pump can ensure the quality of CFRP laminates because the vacuum pump keeps prepregs in the vacuum condition, while the hot press machine provides the steady-state temperature field.

### 2.2. Tensile Test

**Figure 4:**
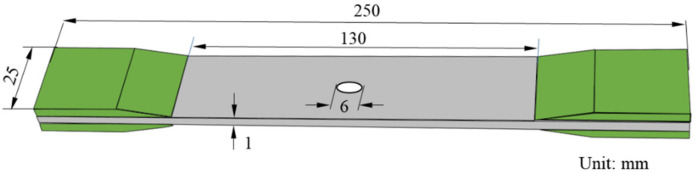
Specimen geometry.

**Figure 5:**
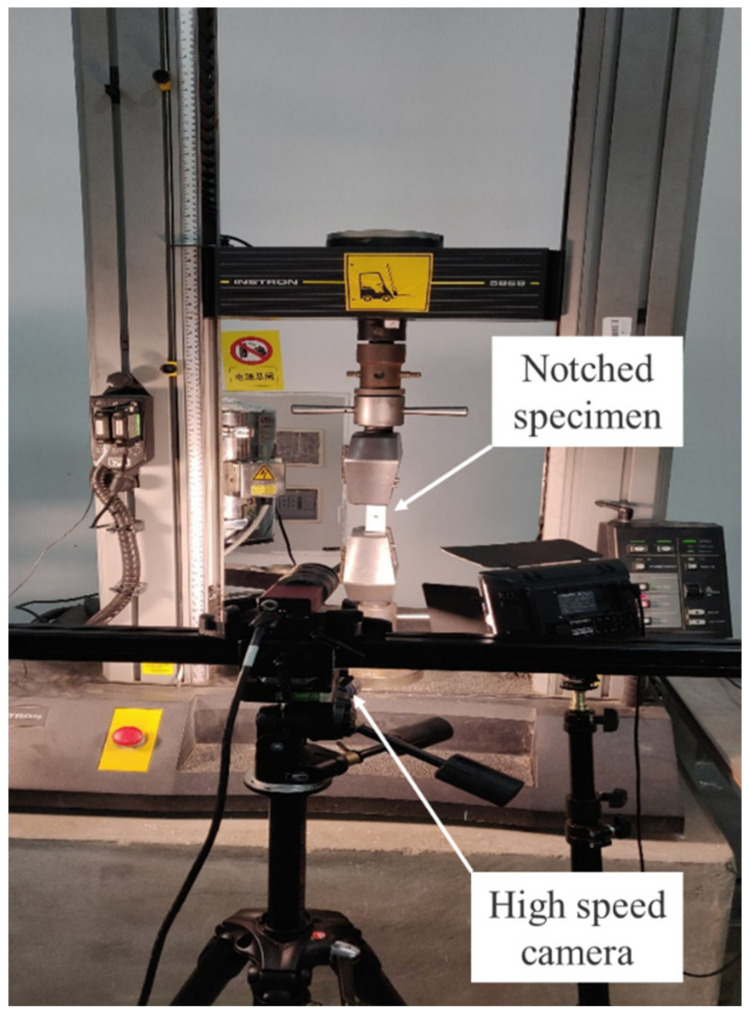
Experimental set-up for digital image correlation (DIC) testing under tension loading.

Specimens for the tensile test with the dimension of 250 mm, 25 mm, and about 1 mm in length, width, and thickness, respectively, were cut from the cured CFRP laminates using a water jet saw. Figure 4 gives the image of the specimen with the gauge length of 130 mm. Glass fiber prepregs were used to fabricate tabs which are bonded to the end of the specimens using two component epoxy paste adhesives. A circular hole with the diameter of 6 mm was drilled at the center of the specimen using a diamond drill bit. Five specimens without the open hole were made for comparative study. Strain gauges with the length of 10 mm were bonded near the open hole. Tensile tests were carried out by an Instron 5869 material testing system with the Instron static load cell, as shown in Figure 5, and the speed of crosshead was set as 0.5 mm/min. In addition, the two-dimensional digital image correlation (DIC) technology was employed to observe the change of displacement and strain of the surface around the open hole. The experimental set-up for DIC testing (Matchid-2D-Stereo, Leuven, Belgium) is shown in Figure 5.

### 2.3. FEM Analysis Model

**Figure 6:**
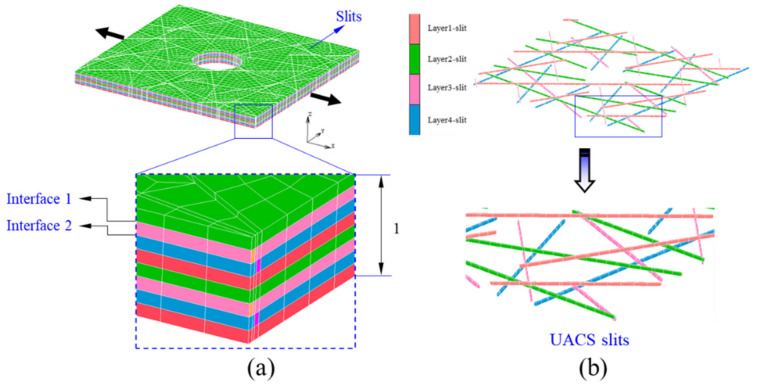
Model for the notched UACS laminate: (**a**) the overall structure of the model, (**b**) detail distribution of slits.

**Figure 7:**
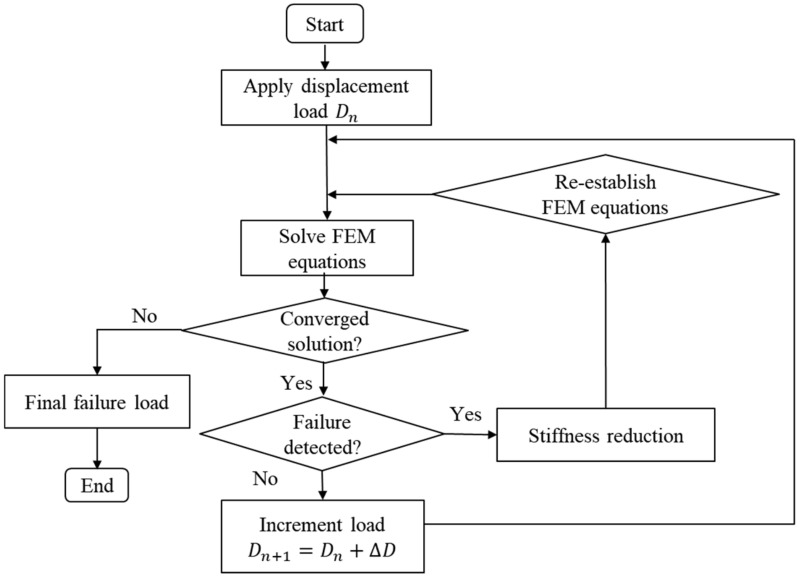
Flowchart of the FEM analysis.

As seen from Figure 3, the distribution of bi-angled slits is very complex. It could be inferred that the micro-structure of the quasi-isotropic UACS laminate is quite complicated. The FEM model as given in Figure 6a, which represents the central region of the specimen with the length, width, and thickness of 25 mm, 25 mm, and 1 mm, respectively. Each layer of the model is represented by the microscopic ply, which actually depicts the distribution of discontinuous slits, as illustrated in Figure 6b. The commercial FEM software of MSC Marc 2012 was selected in this research. As observed from the former experiments [10], slits are filled with the flowing resin during the curing process. Therefore, the material of slits in the model is defined as resin, and the other parts are defined as received CFRP prepreg. In addition, in order to simulate the delamination around 0° ply, cohesive elements are added between 0° ply and ±45° plies. Table 1 gives properties of CFRP prepreg and epoxy resin used in this research, and Table 2 gives material properties of the cohesive element, which shows the bilinear constitute curve. These parameters were provided by the manufacturer of prepreg (Weihai Guangwei) based on the experimental analysis and empirical calculation. The FEM model in the present study is symmetrical. However, it should be noted that the failure usually occurs asymmetrically to the neutral layer of the laminate in tensile tests because the practical laminate could not be perfectly symmetrical due to the existence of microstructural difference. In order to clarify the effect of mesh number on the simulation results, FEM models with four different mesh number (15,264, 23,136, 32,768, 57,288) were performed. It was found that the calculation data tends to converge to a stable value with the increase of mesh number. Considering the calculation cost, all the models were constructed with the number of about 32,000. Solid elements with eight nodes were applied for the model. In this study, the maximum stress criterion was selected in simulating the damage of CFRP and slits. The parameters of the strengths applied in simulation are given in Table 1. Six failure indices *FI_i_* (*i* = 1, 2, … , 6) are defined as shown below:

(1) $$FI_{1}=\frac{\sigma_{1}}{X_{t}},\;FI_{2}=\frac{\sigma_{2}}{Y_{t}},\;FI_{3}=\frac{\sigma_{3}}{Z_{t}},\;FI_{4}=\left|\frac{\sigma_{12}}{S_{12}}\right|,\;FI_{5}=\left|\frac{\sigma_{23}}{S_{23}}\right|,\;FI_{6}=\left|\frac{\sigma_{31}}{S_{31}}\right|,$$

where, *X_t_*, *Y_t_*, and *Z_t_* are tensile strengths of the CFRP in three principal axes, while *S_12_*, *S_23_*, and *S_31_* mean shear strengths in three principal planes of the present material. The detail values of the parameters are listed in Table 1. The displacement load was added at the left edge of the model in the fiber direction of 0^o^ ply, as shown in Figure 6. Figure 7 shows the flowchart of the present damage analysis, adapted from the previous work [11]. It is assumed that all the elements reveal the linear elastic deformation before damage, and the damage diagnosis is conducted at each load step. As for the cohesive element, the damage is following the bilinear property, taking the parameter *d* of 1 as the critical failure criterion. Considering the brittleness of CFRP, once the CFRP failures, the stiffness of the CFRP element decreases to 1% of its initial value. As for slits, linear elastic deformation process is defined before yielding failure in analysis, since the epoxy is isotropic material.

## 3. Results and Discussion

**Figure 8:**
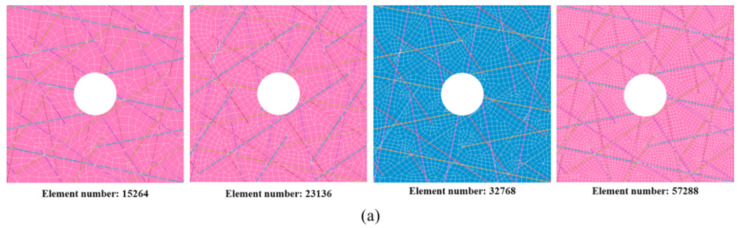

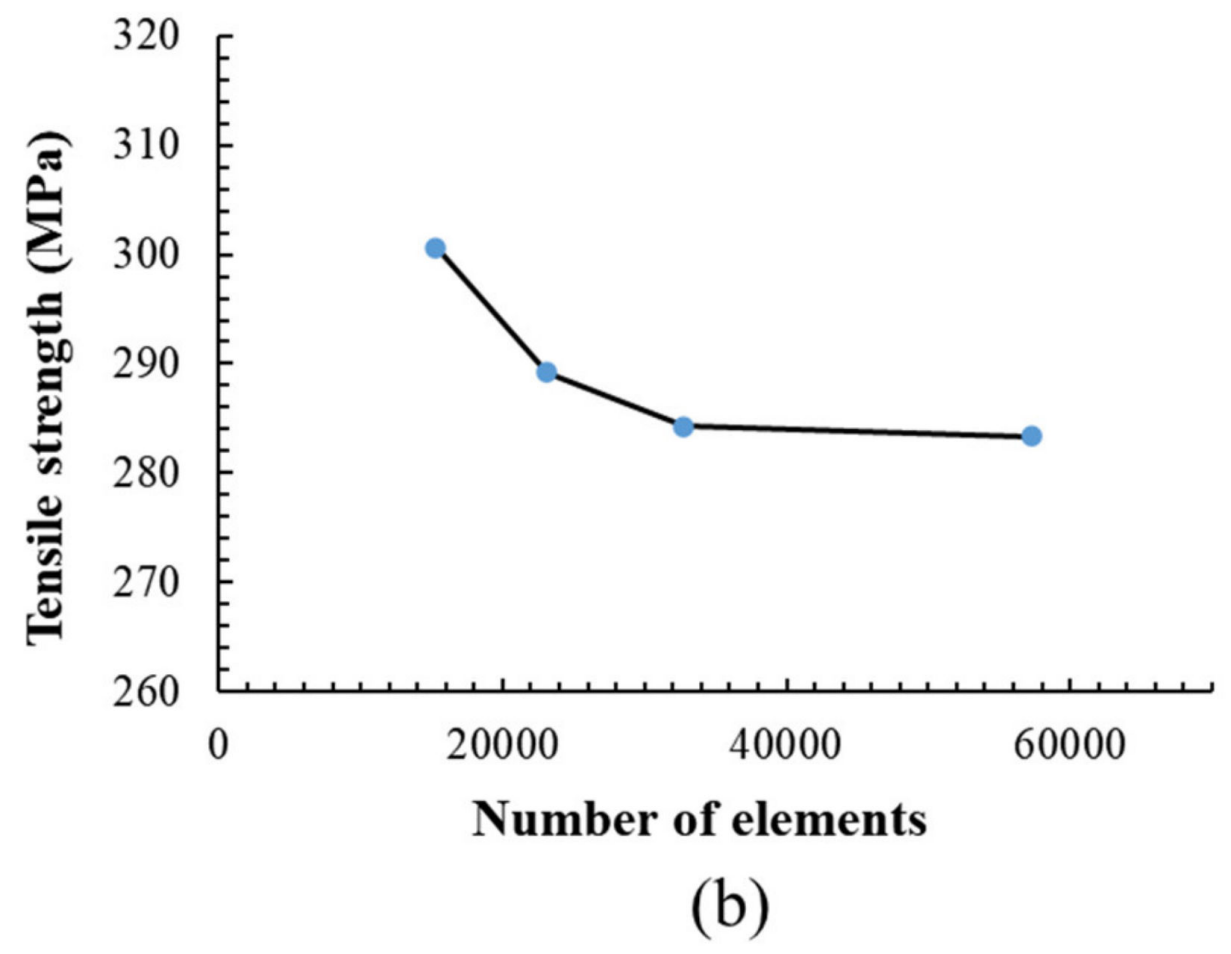
Effect of the mesh number on simulation accuracy: (**a**) FEM model the notched UACS laminates with different element number, (**b**) effect of the element number on the numerical simulation results.

**Figure 9:**
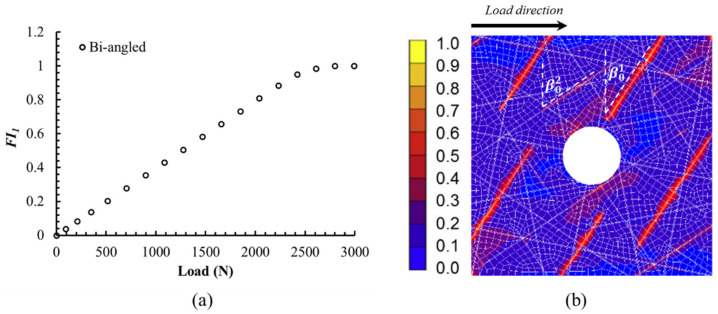
(**a**) Failure index *FI_1_* of the slits in 0° ply with the increase of load, and (**b**) the damage image of the slits in 0° ply at the load of 2.8 kN.

**Figure 10:**
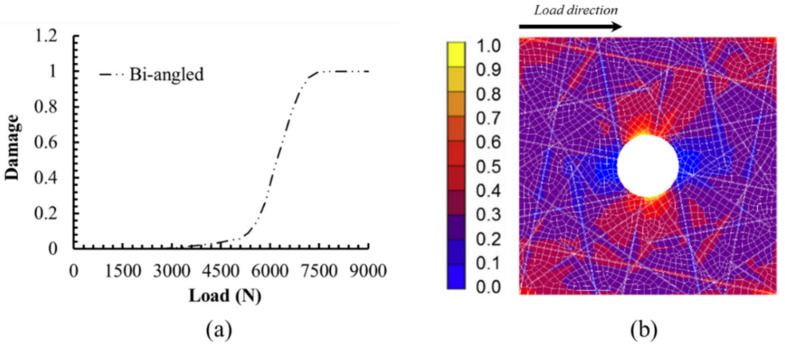
(**a**) The propagation of delamination between the 0° ply and 45° ply with the increase of load from simulation. (**b**) The damage image of the interface 1 at the load of 7.7 kN.

**Figure 11:**
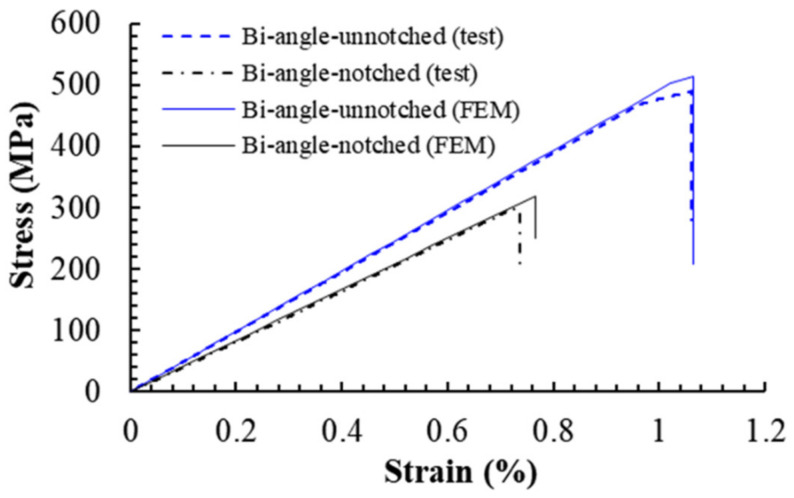
The typical stress-strain curve for the notched and unnotched UACS laminates with bi-angled slits obtained from numerical simulation and experiments.

**Figure 12:**
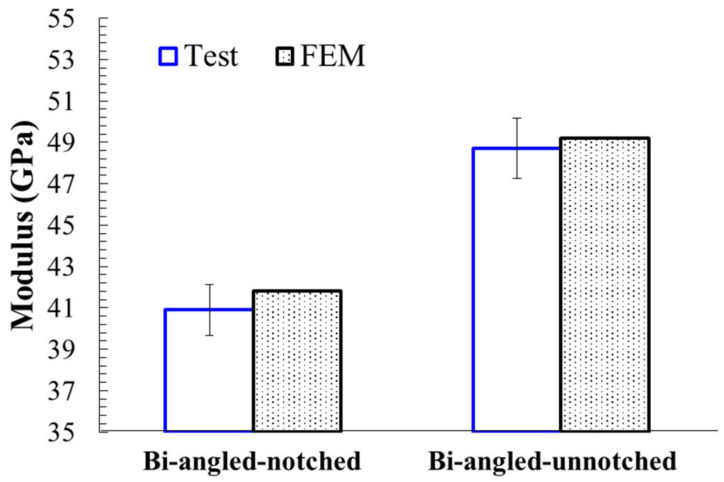
Modulus of the notched and unnotched UACS laminates with bi-angled slits obtained from numerical simulation and experiments.

**Figure 13:**
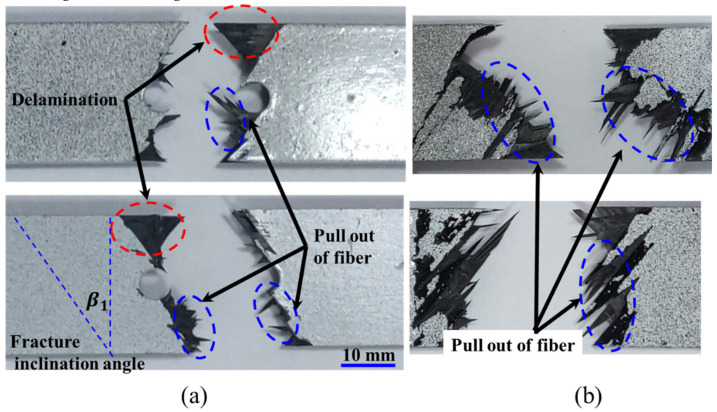
Images of fractured specimens of UACS laminates with bi-angled slits: (**a**) notched UACS laminates, (**b**) unnotched UACS laminates.

**Figure 14:**
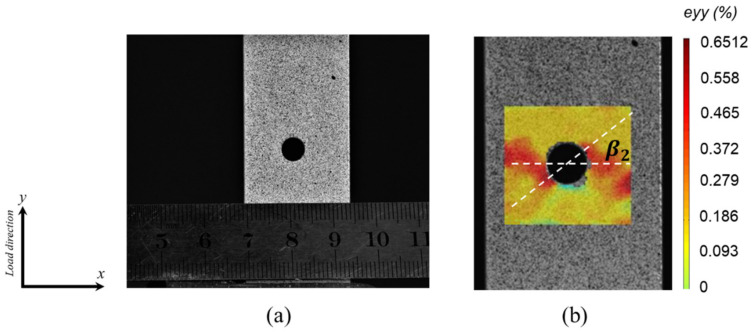
Typical DIC strain field near the open hole of the notched specimen: (**a**) specimen surface with random speckle pattern, (**b**) typical strain field in the load direction of the present notched UACS laminate at 90% of the final failure load (7.1 kN).

Obtained numerical and experimental results are listed in Figure 8, Figure 9, Figure 10, Figure 11, Figure 12, Figure 13, Figure 14 and Table 3. Figure 8 shows the effect of the mesh number on simulation accuracy. FEM models with four kinds of mesh number are given in Figure 8a, and it can be easily imaged that the mesh become denser with the increasing of mesh number. From Figure 8b, it could be found that the strength converges to a stable level when the mesh number is over about 30,000. It is inferred that the mesh number of about 32,000 is suitable for the present simulation based on these results, which can ensure good accuracy and reduce a certain computational cost. Therefore, all the models used in the present study were constructed with the mesh number of about 32,000. Furthermore, models with similar structure and mesh number of unnotched UACS laminate were constructed for comparative study.

Failure loads for different stages of crack propagation of notched and unnotched UACS laminates with bi-angled slits are given in Table 3. The first failure is found in the slits of 0° plies near the hole at relatively low load of about 2.8 kN for the notched laminate, which is about 35% of the final failure load. After that, failures are found in the matrix of 90° plies and ±45° plies at loads of 3.7 kN and 5.1 kN, respectively. Then, the delamination occurs between the 0° ply and ±45° plies at the load of 7.7 kN, which is quite close to the final failure load of 7.9 kN, because there are a few fiber breakages in the case of notched UACS laminate with bi-angled slits. It could be found that the maximum load of notched UACS laminate is about 60% of that of the unnotched one. Furthermore, the load level at different stages of the damage propagation process for the notched UACS laminate is lower than that for the unnotched one, and it is considered to be caused by the stress concentration around the open hole. Figure 9a shows the progressive failure curve of index *FI_1_* of slits in the 0° ply with the increase of load. It is clear that the failure index *FI_1_* increases linearly before final failure, and it could be considered as the elastic deformation before yielding. Figure 9b gives the damage image of slits in the 0° ply at 2.8 kN. It could be clearly seen that the initial damage occurs mainly along slits with the inclination angle of $\beta_{0}^{1}$ = 33.7° instead of $\beta_{0}^{2}$ = 56.3° due to the larger normal stress at slits of $\beta_{0}^{1}$.

Figure 10a depicts the propagation of delamination between the 0° ply and 45° ply with the increase of load from simulation. The vertical axis means the damage parameter *d* of the cohesive element as defined in Section 2.3. It could be seen that the damage parameter increases quite slowly at loads less than 5 kN. After that, the damage parameter increases rapidly until the final failure. As seen from Table 3, it could be understood that damages have existed in the matrix of 90° and ±45° plies at the load of 5 kN. Therefore, it is inferred that the failure in the matrix of ±45° plies plays a significant role in accelerating the propagation of delamination between 0° and its adjacent plies. In addition, the initial damage mainly appears near the open hole due to the stress concentration, as illustrated in Figure 10b. Based on the FEM simulation, it is concluded that the damages of the UACS laminate under tensile load initiates from slits of 0° ply, then propagates to the 90° and ±45° plies, and the final failure damage is mainly dominated by the delamination. These results are obtained according to the variation of *FI_1_*, *FI_2_*, and *d* of the model. It was found that parameters from *FI_3_* to *FI_6_* show little effect on the damage of notched UACS laminate, which is consistent with the previous observation [11].

Typical stress-strain curves for notched and unnotched UACS laminates with bi-angled slits obtained from numerical simulation and experiments are depicted in Figure 11. It can be seen that, for the unnotched UACS laminate, both experiment and simulation captured the nonlinear behavior before the final failure, which is considered to be the nonlinear progressive damage in matrix and delamination. The simulation results overall match well with the experimental result, although there is a little overestimate on strength for the simulation by about 5% compared to the experiment. As for the notched UACS laminate, the tensile strength decreased dramatically to about 60% of the strength of unnotched UACS laminate. A similar overestimate on strength (7%) for the simulation was observed. It is clear that the present simulation method shows better accuracy in simulating the unnotched UACS laminate compared to the notched laminate, both for strength and strain. It is actually reasonable because it is inevitable that there will be minor damage at the free edge during cutting, especially damage around the open hole during the process of drilling; therefore, it is difficult to prepare perfect notched and unnotched specimens in an experiment, like the FEM models in simulation. In addition, no nonlinear behavior is observed in the stress-strain curves of notched UACS laminates, both those obtained from experiments and simulation. It means that, for the notched UACS laminate, the damage propagated very quickly before final failure compared to unnotched ones, due to the significant stress concentration near the open hole, while Figure 12 gives the modulus of the notched and unnotched UACS laminates with bi-angled slits obtained from numerical simulation and experiments. The modulus of the notched UACS laminate is 40.9 GPa, which is about 84% of the unnotched one. Obviously, the present analysis model shows excellent accuracy in simulating modulus with the error less than 3%.

Figure 13 shows typical images of fractured specimens of notched and unnotched UACS laminates with bi-angled slits. As for the notched UACS laminate, a large region of delamination could be found from the fracture surface as marked by the red ellipse dashed lines, and there is relatively less fiber pulling out phenomenon as depicted by the blue ellipse dashed lines, which clearly differs from the fracture pattern of the unnotched UACS laminate with bi-angled slits as shown in Figure 13b. It is understood that the final critical failure mode for the notched UACS laminate is mainly dominated by the delamination instead of the fiber breakage in unnotched UACS laminates. This experimental observation shows good agreement with simulation results described in Table 3. In addition, the average fracture inclination angle $\beta_{1}$ of notched UACS specimens is measured as about 35.2°, which is quite close to the difference between the ply angle (45°) and inclination angle of slits (11.3°). Therefore, it is inferred that the notched UACS laminate mainly fractures along the slits in ±45° after the initial failure in slits and the consequent delamination, as shown in Figure 9b and Figure 10b. 

The DIC technology is used to investigate the strain field around the open hole of the notched specimen, and the typical image is given in Figure 14. Figure 14a shows the specimen surface with random speckle pattern, which is used to track the deformation of the specimen surface, while the typical strain field in load direction of the present notched UACS laminate at 90% of the final failure load (7.1 kN) is presented in Figure 14b. It is observed that the high strain concentration in load direction occurred around the open hole, especially along the inclination angle $\beta_{2}$, which is measured in the range from 32° to 38°. It shows good agreement with the observation in Figure 9 and Figure 13. Under this load, failure has already existed in the matrix of all the plies; however, the delamination does not yet occur, according to Table 3.

## 4. Conclusions

In the present study, UACS laminates were fabricated using the continuous CFRP prepreg, on which regularly arrayed bi-angled slits were introduced by a commercial numerical control cutter. The tensile strength and fracture morphology of notched UACS laminates were experimentally studied. while the tensile strength and modulus, critical failure loads for different types of damages, and the progressive failure process of the notched UACS laminate were investigated by numerical simulation. The main conclusions are listed as follows:

(1) The tensile strength of notched UACS laminate is measured as 298 MPa, which is about 60% of the strength of unnotched UACS laminate. The modulus of the notched UACS laminate is 40.9 GPa, which is about 84% of the unnotched one.
(2) A large region of delamination is observed from the fracture surface of notched UACS laminate, and there is relatively less fiber pulling out phenomenon compared to the fracture pattern of unnotched UACS laminate. The final critical failure mode for the notched UACS laminate is mainly dominated by the delamination instead of the fiber breakage in unnotched UACS laminates.
(3) Simulation results match well with the experiments, although there is a little overestimate on strength by about 5% and 7% for the unnotched and notched UACS laminates, respectively, compared to the experiment. The present analysis model shows excellent accuracy in simulating the modulus, with the error less than 3%.
(4) The simulation reveals the critical failure loads for different types of damages in the progressive failure process for the notched UACS laminate. The failure initiates from slits of 0° plies near the hole at a relatively low load of about 2.8 kN. After that, failures occur in the matrix of 90° plies and ±45° plies at loads of 3.7 kN and 5.1 kN, respectively. The delamination occurs between the 0° ply and ±45° plies at the load around 7.7 kN. Then, the final failure is quickly after that, at the load of 7.9 kN.

## Figures and Tables

**Table 1:** Properties of CFRP and epoxy resin.

CFRP	
Longitudinal Young’s modulus *E*_1_ (GPa)	139
Transverse Young’s modulus *E*_2_=*E*_3_ (GPa)	8.8
In-plane shear modulus *G*_12_ *=G*_31_ (GPa)	4.2
Out-of-plane shear modulus *G*_23_ (GPa)	3.7
In-plane Poisson’s ratio	0.27
Out-of-plane Poisson’s ratio	0.3
Longitudinal tensile strength *X*_t_ (MPa)	2900
Longitudinal compression strength *X*_c_ (MPa)	1600
Transverse tensile strength *Y*_t_ =*Z*_t_ (MPa)	80
Transverse compression strength *Y*_c_=*Z*_c_ (MPa)	190
In-plane shear strength *S*_12_ *=S*_31_ (MPa)	140
Out-of-plane shear strength *S*_23_ (MPa)	90
Epoxy resin	
Young’s modulus *E* (GPa)	3.8
Poisson’s ratio	0.32
Tensile strength (MPa)	80
Compression strength (MPa)	190
Shear strength (MPa)	90

**Table 2:** Properties of the cohesive interface element.

Cohesive Element	
Critical energy release rate *G_c_*	0.32 N/mm
Critical opening displacement *d_c_*	5.6×10^-6^ mm
Maximum opening displacement *δ_m_*	0.012 mm

**Table 3:** Failure loads for different stages of the damage propagation.

Failure Loads (kN)	Bi-Angled	
	Unnotched	Notched
Failure in slits of 0^o^ plies (*FI_1_*=1)	4.9	2.8
Failure in matrix of 90 ^o^ plies (*FI_2_*=1)	6.9	3.7
Failure in matrix of ±45 ^o^ plies (*FI_2_*=1)	9.8	5.1
Delamination (*d* = 1)	11.8	7.7
Final fracture	12.4	7.9
